# Heterotrophy mitigates the response of the temperate coral *Oculina arbuscula* to temperature stress

**DOI:** 10.1002/ece3.2399

**Published:** 2016-08-31

**Authors:** Hannah E. Aichelman, Joseph E. Townsend, Travis A. Courtney, Justin H. Baumann, Sarah W. Davies, Karl D. Castillo

**Affiliations:** ^1^ Department of Marine SciencesUniversity of North Carolina at Chapel HillChapel HillNorth Carolina; ^2^ Department of Marine and Environmental SciencesNortheastern UniversityNahantMassachusetts; ^3^ Present address: Scripps Institution of OceanographyUniversity of CaliforniaSan DiegoLa JollaCalifornia

**Keywords:** Bleaching, climate change, coral, heterotrophy, *Oculina arbuscula*, temperate

## Abstract

Anthropogenic increases in atmospheric carbon dioxide concentration have caused global average sea surface temperature (SST) to increase by approximately 0.11°C per decade between 1971 and 2010 – a trend that is projected to continue through the 21st century. A multitude of research studies have demonstrated that increased SSTs compromise the coral holobiont (cnidarian host and its symbiotic algae) by reducing both host calcification and symbiont density, among other variables. However, we still do not fully understand the role of heterotrophy in the response of the coral holobiont to elevated temperature, particularly for temperate corals. Here, we conducted a pair of independent experiments to investigate the influence of heterotrophy on the response of the temperate scleractinian coral *Oculina arbuscula* to thermal stress. Colonies of *O. arbuscula* from Radio Island, North Carolina, were exposed to four feeding treatments (zero, low, moderate, and high concentrations of newly hatched *Artemia* sp. nauplii) across two independent temperature experiments (average annual SST (20°C) and average summer temperature (28°C) for the interval 2005–2012) to quantify the effects of heterotrophy on coral skeletal growth and symbiont density. Results suggest that heterotrophy mitigated both reduced skeletal growth and decreased symbiont density observed for unfed corals reared at 28°C. This study highlights the importance of heterotrophy in maintaining coral holobiont fitness under thermal stress and has important implications for the interpretation of coral response to climate change.

## Introduction

Anthropogenic activities have increased global atmospheric carbon dioxide (*p*CO_2_) from approximately 280 ppm during the Industrial Revolution to present‐day values exceeding 400 ppm (Doney et al. 2009). This increase in atmospheric *p*CO_2_ has resulted in global sea surface temperature (SST) increases of 0.11°C per decade between 1971 and 2010, and these trends are projected to continue into the 21st century (Rhein et al. 2013). Notably, over the last several decades, warming has been more prominent in the North Atlantic Ocean relative to other ocean basins with warming of up to 4°C predicted for temperate Atlantic waters by the end of this century (Rhein et al. 2013). This ocean warming trend has affected the health of marine ecosystems, including thermally sensitive coral reef habitats worldwide (Hoegh‐Guldberg 1999; Hoegh‐Guldberg and Bruno 2010). Tropical corals are stenothermal, that is they can tolerate a small range of temperatures, and thus even small changes in seawater temperature can result in “coral bleaching” – the loss of corals' photosynthetic endosymbionts (*Symbiodinium* spp.) and/or photosynthetic pigments – which in turn can negatively affect corals, including by reducing their growth and calcification (Jokiel and Coles 1990; D'Croz et al. 2001; Hoegh‐Guldberg et al. 2007; Donner 2009). Although the effects of rising SST on tropical corals are well investigated (Lesser 1997; Hoegh‐Guldberg and Bruno 2010; Hughes et al. 2010; Doney et al. 2012), uncertainty remains as to how temperate corals have responded to recent warming and how they will cope with predicted end‐of‐century ocean warming (Wernberg et al. 2011; Verges et al. 2014).

In many temperate coastal environments, including the North Carolina (NC) coast, SSTs have increased concurrently with other anthropogenic stressors (Paerl et al. 2006). Human development along NC's coastal watersheds has increased nutrient loading, leading to eutrophication (Paerl et al. 2003, 2006). This eutrophication has triggered changes in primary and secondary production, altering the trophic structure of NC coastal ecosystems (Paerl et al. 2003). Changes in primary and secondary production have the potential to affect corals as they obtain carbon (C) and nutrients not only from the photosynthetic byproducts of their endosymbiotic algae (photoautotrophy), but also by feeding on plankton and dissolved/particulate matter from the water column (heterotrophy; Houlbreque and Ferrier‐Pages 2009). Generally, photoautotrophic C is used for metabolic demands and calcification, while heterotrophic C is allocated for building tissue and growth (Hughes et al. 2010). Coral heterotrophy also supplements nutrients not provided by coral endosymbionts through the capture of dissolved organic matter, particulate organic matter, and zooplankton (Houlbreque and Ferrier‐Pages 2009). Photoautotrophy can provide up to 100% of a coral's daily metabolic requirements; therefore, when corals bleach and lose symbiont‐derived C, they must either reduce metabolic demand, rely on existing energy reserves, or increase heterotrophy (Grottoli et al. 2006; Levas et al. 2013).

Previous studies have shown that heterotrophy allows some species of tropical corals to mitigate the negative effects of thermal stress and ocean acidification (OA), which include but are not limited to decreased photosynthetic activity, loss of pigmentation, and reduced calcification (Grottoli et al. 2006; Borell et al. 2008; Cohen and Holcomb 2009; Edmunds 2011). For example, research has demonstrated the importance of heterotrophy in the tropical scleractinian coral *Montipora capitata*, which met up to 100% of its daily metabolic requirements by increasing feeding rates when recovering from a bleaching event (Grottoli et al. 2006). More recently, it was shown that the negative effects of OA and temperature stress in *Acropora cervicornis* were mitigated by increased feeding rates (Towle et al. 2015). Taken together, these studies suggest that heterotrophic feeding is one method by which tropical corals can cope with stressors associated with climate change; however, this effect is understudied in temperate coral species.

*Oculina arbuscula* is a facultatively symbiotic coral, meaning that it can exist as a healthy colony both with (symbiotic) or without (aposymbiotic) its endosymbionts (Miller 1995). Aposymbiotic colonies of *O. arbuscula* use zooplankton of the pico‐ and/or nanoplankton size class (<63 μm) almost exclusively to obtain nutrition, while symbiotic colonies of *O. arbuscula* rely almost entirely on C translocated from their endosymbionts to meet metabolic demands (Leal et al. 2014). Therefore, the trophic ecology of this temperate coral may be more complicated than its tropical coral counterparts, which primarily exhibit obligate symbiosis. To date, no studies have investigated the physiological response of C acquisition in *O. arbuscula* under thermal stress; however, previous research has investigated the impact of heterotrophy and temperature on growth and symbiont density in other temperate coral species.

Recent studies on temperate corals have revealed a positive relationship between temperature and coral growth (i.e., coral growth increases with increasing temperature;Jacques et al. 1983; Miller 1995) as well as a positive relationship between feeding and growth (Kevin and Hudson 1979; Miller 1995) and photosynthesis of algal symbionts (Szmant‐Froelich and Pilson 1984; Piniak 2002). Growth rates of the symbiotic temperate scleractinian coral *Cladocora caespitoa* have been shown to be driven primarily by increased temperature and heterotrophic food supply (Rodolfo‐Metalpa et al. 2008). However, when the same species was exposed to temperatures 4°C above normal maximum summer temperature (28°C), the result was tissue necrosis that eventually led to bare skeleton and coral fragment death (Rodolfo‐Metalpa et al. 2005). Recently, these results were contrasted by a study which demonstrated that long‐term exposure of *C. caespitosa* to thermal stress (29°C) resulted in no impact on tissue necrosis or photosynthetic efficiency (Kersting et al. 2015). However, exposure to temperature stress in addition to the presence of invasive algae negatively impacted photosynthetic efficiency and caused tissue necrosis in the coral (Kersting et al. 2015). Another study found that for two temperate coral species (*C. caespitosa* and *Oculina patagonica*), short‐term thermal stress up to 5°C above the mean summer temperature resulted in no change in symbiont density (Rodolfo‐Metalpa et al. 2006). Taken together, these studies demonstrate the variable manner in which temperate corals respond to thermal stress and illustrate the need for a more comprehensive understanding of how temperate corals are likely to respond to future climate change.

**Figure 1:**
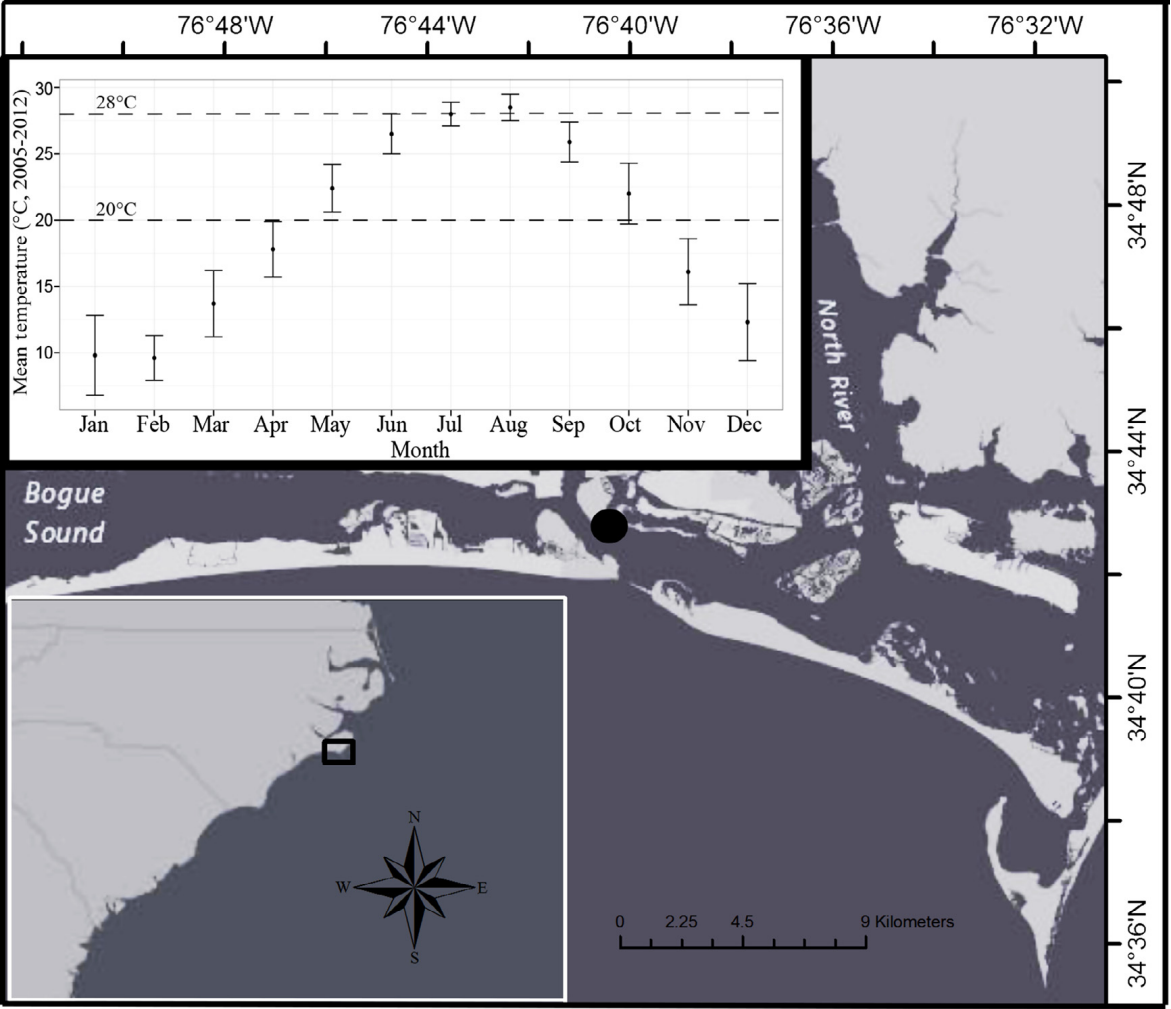
Location and mean temperatures of collection site. Map showing the location where the *Oculina arbuscula* colonies were collected at Radio Island, North Carolina (indicated by a black dot). The inset graph shows average monthly sea surface temperature for 2005–2012 from NOAA buoy BFTN7 located 1.3 km from collection site. Error bars represent standard deviation. Dotted lines on the inset graph at 20 and 28°C indicate the yearly average temperature (mild stress experiment) and the approximate average summer temperature (moderate stress experiment), respectively.

The aim of this study was to determine the effect of heterotrophy on growth and symbiont density of the temperate coral *O. arbuscula* under thermal stress. The temperate scleractinian coral *O. arbuscula* was selected for this study because its environment is experiencing changes in primary and secondary production (Paerl et al. 2006) as well as variations in SST (Rhein et al. 2013). *Oculina arbuscula* inhabits the southeastern and mid‐Atlantic US up to 200 m depth (Miller 1995) and helps create hard‐bottom structure that supports economically valuable fisheries species and a variety of other ecologically and economically important organisms (Deaton et al. 2010). *Oculina arbuscula* were fed four concentrations of freshly hatched *Artemia* sp. nauplii based on representative field concentrations of plankton quantified at the collection site (Fulton 1984). Corals were reared under feeding conditions for approximately 40 days at the average annual temperature (mild stress: 20°C) and average summer temperature (moderate stress: 28°C) of the collection site (Fig. 1), and the effects of heterotrophy on growth and symbiont density under thermal stress were investigated. Understanding how *O. arbuscula* responds to temperature stress will allow for better predictions of how coral‐dominated benthic hard‐bottom ecosystems may shift in the face of a changing climate and will help to inform environmental management decisions in the Southeast US.

## Materials and Methods

### Collection and transportation

In February 2014, 12 10‐ to 15‐cm‐diameter symbiotic colonies of *O. arbuscula* were collected at Radio Island Jetty near Beaufort, NC, using a hammer and chisel (Fig. 1). All *O. arbuscula* colonies were collected under the NC Division of Marine Fisheries Permit #706481. During collection, seawater temperature (8.50 ± 0.01°C; mean ± SE) was measured every 10 min using a HOBO Water Temperature Pro V2 Logger (Onset, Bourne, MA). *Oculina arbuscula* colonies were collected at a depth of approximately 3 m across a linear distance of ~200 m. Colonies collected were separated by at least 5 m in an effort to avoid the collection of identical genotypes. Colonies were transported to the Aquarium Research Center at the University of NC at Chapel Hill where corals were maintained in four 500‐L recirculating holding tanks at approximately ambient field temperature (9.4 ± 0.1°C; ±SE) and salinity (35.2 ± 0.04; ±SE).

Temperature treatments were determined based on 2005–2012 data from a NOAA buoy (station BFTN7), located approximately 1.3 km from the collection site. The mild thermal stress experiment (20°C) represents the mean annual temperature measured at the NOAA buoy between January 2005 and December 2012 (20.0 ± 0.01°C, ±SE). The moderate thermal stress experiment (28°C) represents the approximate average summer temperature (June–August) measured for the same time interval as above (27.7 ± 0.6°C, ±SE; NOAA National Data Buoy Center; Fig. 1). The moderate thermal stress experiment (28°C) reflects a chronic exposure to elevated temperature conditions relative to ambient seawater temperature conditions at the time of collection. Due to the contrast between the temperature at collection (approximately 9°C) and the experimental temperatures (20°C or 28°C), we refer to the two thermal experiments as “mild stress” and “moderate stress.” Due to tank limitations and logistical concerns, the two thermal experiments (20 and 28°C) were not run concurrently. Each thermal experiment was therefore considered statistically mutually exclusive in all analyses. This is because each thermal experiment was conducted on unique genotypes and experienced different pretreatment conditions.

### Recovery and acclimation

#### Mild temperature stress experiment (20°C)

Half of the 12 *O. arbuscula* colonies (*N* = 6) collected were assigned to the mild temperature stress experiment (20°C). Each mild stress *O. arbuscula* colony was sectioned into 12 fragments using a diamond‐embedded band saw (Inland, Madison Heights, MI) and mounted on sterile plastic petri dishes using cyanoacrylate. This sectioning yielded a total of 72 approximately equal‐sized fragments (12 per colony), each weighing between 15 and 25 g wet weight. Fragments were given approximately 1 week for recovery, after which pre‐acclimation buoyant weight measurements were conducted. Coral fragments were maintained at ambient field temperature for a total of 10 days after collection, at which point temperatures were slowly increased by approximately 0.5°C per day until the 20°C target temperature was achieved. All colonies were fed equally at the moderate concentration of newly hatched *Artemia* sp. nauplii (250 *Artemia* sp. nauplii per L) and were acclimated at 20°C until corals were assigned to their respective feeding treatments for the duration of the 38‐day feeding experiment.

#### Moderate temperature stress experiment (28°C)

The other half of the *O. arbuscula* colonies (*N* = 6) remained as full colonies at ambient field temperatures for 10 days after collection and were increased to 20°C along with the mild stress experiment colonies. Moderate stress colonies were maintained at 20°C for 23 days, at which point colonies were sectioned using methods described above, which produced 72 approximately equally sized fragments (12 per colony). Colonies used in the moderate stress experiment were maintained at 20°C until completion of the mild stress experiment (20 days), after which time fragments were placed in experimental tanks and temperatures were slowly increased by approximately 0.5°C per day until the target 28°C was achieved. All colonies were fed equally at the moderate concentration of *Artemia* sp. nauplii (250 *Artemia* sp. nauplii per L) and allowed to acclimate to 28°C until the corals were assigned to feeding treatments for the duration of the 37‐day feeding thermal stress experiment.

### Experimental design

Mild and moderate temperature stress experiments were conducted using the same experimental seawater system consisting of 12 38‐L experimental aquaria. Three aquaria were assigned to each of four feeding treatments (zero, low, moderate, and high). For each thermal experiment, *O. arbuscula* fragments from six colonies (6 colonies × 12 fragments = 72 fragments) were placed in aquaria such that each genotype was represented in all four of the heterotrophic feeding regimes (*n* = 6 fragments per aquarium).

Each feeding treatment consisted of three 38‐L aquaria connected to a 190‐L sump. Feeding treatments shared a high‐output T5 lighting system (Current‐USA, Vista, CA) containing two 460‐nm actinic bulbs and two 10,000‐K daylight bulbs (156 watt fixture). To simulate dawn and dusk, corals were only exposed to actinic lights for the first and last hours of the 12‐h light cycle. This lighting system maintained an average photosynthetically active radiation (PAR) of approximately 300 μmol photons·m^−2^·sec^−1^ at the base of each aquarium. Experimental PAR conditions were based on spot measurements made during collection (approximately mid‐day on 8 February 2014) that ranged between 200 and 400 μmol photons·m^−2^·sec^−1^ as well as on values used in previous tank experiments for the same species and collection site (Miller 1995). Each aquarium contained two powerheads (Hydor USA, Sacramento, CA) rated at 908.5 Lh^−1^. The sump filtration system consisted of a filter sock to remove particulates and a protein skimmer (Eshopps, City of Industry, CA) to remove organic materials, both of which were regularly cleaned following feeding. Each aquarium was covered with a transparent plexiglass sheet to limit evaporative water loss. Mild stress treatments were maintained at 20 ± 0.1°C (±SE) by circulating the seawater through a chiller (AquaEuroUSA, Los Angeles, CA). Moderate stress treatments were maintained at 27.9 ± 0.1°C (±SE) using 50‐W heaters (Eheim, Deizisau, Germany).

For both experiments, *O. arbuscula* were fed newly hatched *Artemia* sp. nauplii three times a week at their respective treatment concentrations. *Artemia* sp. nauplii concentration for the moderate feeding treatment was determined from average copepod abundance measured near Beaufort, NC (8414 copepods·m^−3^ or ~250 *Artemia* sp. nauplii per L; Fulton 1984). Low and high feeding treatments were calculated as half and twice the moderate concentration, respectively. The four feeding treatment target concentrations were approximately (1) zero: 0 *Artemia* sp. nauplii per L; (2) low: 125 *Artemia* sp. nauplii per L; (3) moderate: 250 *Artemia* sp. nauplii per L; and (4) 500 *Artemia* sp. nauplii per L. The amount of *Artemia* sp. nauplii added to each feeding treatment was estimated by counting in triplicate the number of hatched nauplii in 1 mL of water and extrapolating to the concentrations listed above. For example, an average hatch contained approximately 100 *Artemia* sp. nauplii per mL, and in order to obtain 250 *Artemia* sp. nauplii per L (7650 *Artemia* sp. nauplii per aquaria) in the moderate feeding system, 153 mL of hatched *Artemia* sp. nauplii was added.

Each recirculating sump system and protein skimmer were turned off before feeding commenced in order to isolate individual aquarium during feeding. Feeding began at least 30 min after aquarium lights were turned off (12‐h day–night cycle) to simulate crepuscular feeding. After the aquaria were isolated, the respective amounts of newly hatched *Artemia* sp. nauplii were added to each aquarium. Powerheads were left on in every aquarium during feeding to ensure circulation of the *Artemia* sp. nauplii. Aquaria remained isolated and corals were allowed to feed for 1 h. In order to limit positional effects across *O. arbuscula* fragments within an aquarium, fragments were rotated prior to each feeding event. At the completion of the mild and moderate temperature stress experiments, each *O. arbuscula* fragment was photographed and had its tissue airbrushed. The tissue slurry was then frozen at −20°C, and the remaining coral skeletons were dried overnight at 50°C in a drying oven (Quincy Lab, Chicago, IL).

### Aquarium conditions

Seawater salinity was formulated to 36.00 ± 0.07 (±SE) using *Instant Ocean Sea Salt* mixed with deionized water. Compared to other commercially available seawater mixes, *Instant Ocean Sea Salt* is most similar to natural seawater in its major and minor elemental composition as well as its carbonate chemistry (Atkinson and Bingman 1998). Deionized water was added between water changes to account for evaporation, and 50% water changes were performed weekly across all aquaria. Nitrate (${\text{NO}}_{3}^{-}$) concentrations were monitored weekly in all aquaria using an Aquarium Pharmaceuticals Nitrate Test Kit (API, Chalfont, PA) to ensure that there were no excess nutrients in the experimental aquaria. Each measurement found negligible concentrations of ${\text{NO}}_{3}^{-}$ in all experimental aquaria. Seawater chemistry, including salinity, temperature, and pH, were monitored and recorded before each feeding and corrected as necessary. Salinity was measured using a YSI 3200 conductivity meter, temperature was measured using a NIST‐calibrated partial‐immersion organic‐filled glass thermometer, and pH was measured using an Orion Star A211 pH meter with a ROSS Sure‐Flow Combination pH probe calibrated with certified NBS pH buffers of 4.01, 7.00, and 10.01. All data for tank parameters are available in Table S1.

### Quantification of calcification rates and symbiont density

*Oculina arbuscula* calcification rates were estimated using the buoyant weight method with a bottom‐loading balance (precision = 0.0001 g; Mettler‐Toledo, Columbus, OH;Davies 1989). Buoyant weight measurements (*N* = 3 replicate measurement/fragment) were taken before acclimation and at the start and end of each temperature experiment. Because buoyant weight measurements occurred over several days at each time point, growth data were corrected for the number of days in each experimental interval.

Symbiont counts were completed using the hemocytometer method (Rodrigues and Grottoli 2007). Airbrushed samples were thawed and homogenized for 5 min using a Tissue‐Tearor (Dremel, Racine, WI). Samples were then centrifuged for 15 min at 3030 g. Equal volumes of formalin and Lugol's iodine were added to the pellets, which were homogenized to re‐suspend the pellet. Three replicates of 10 μL subsamples were counted from the stained symbiont suspensions on a hemocytometer using a light microscope. Technical replicates for symbiont counts from the three subsamples were then averaged and normalized to the volume of the tissue slurry as well as the surface area of the corresponding nubbin.

After drying overnight at 50°C, coral skeletons were weighed to determine dry weights. Final buoyant weights and dry weights for coral fragments were linearly correlated (Fig. S1, *R* ^2^ = 0.997) with the following equation describing this relationship:

Dry Weight(mg)=1.591×Buoyant Weight(mg)+4105.6.

Using this equation, initial dry weight was estimated from initial buoyant weight. This proxy allowed for the expression of coral growth as the change in dry weight, or net calcification (Ries et al. 2009; Courtney et al. 2013). Organic materials that attach to the coral tissue and have a density different from that of seawater may influence buoyant weight measurements. For this reason, we chose to use dry weight and express coral growth as net calcification instead of percent change in buoyant weight (Ries et al. 2010).

Surface area of *O. arbuscula* fragments was calculated using a 3D laser scanner (NextEngine, Santa Monica, CA) and was used to normalize buoyant weight and symbiont counts for each coral fragment to unit area (mg·cm^−2^ or cells·cm^−2^, respectively). Normalizing to surface area corrects for areas over which new skeleton was deposited over the experimental interval (Elahi and Edmunds 2007).

### Statistical analyses

All statistical analyses were implemented using R software, version 3.0.1 (R Development Core Team, 2015). Analyses of variance (ANOVA, function *aov()*) were used to determine the effects of feeding and genotype on the difference in dry weight and the change in symbiont density (normalized to surface area) across each thermal experiment. To meet ANOVA assumptions, symbiont densities were log‐transformed and all models were tested for equal variance of the residuals and normality (Fig. S2). For both thermal experiments, two fixed factors were modeled: feeding treatment and genotype, with tank nested within feeding treatment. Genotype was included as a fixed effect to minimize the type 1 errors; however, variations in genotypes were not the focus of the study. If factors were found to be significant (*P* < 0.05), post hoc Tukey's HSD tests were used to evaluate the significance of each pairwise comparison. Data for plotting were produced using the function *summarySE()* and plotted implemented in the package *ggplot2*. All R scripts and data collected in this experiment are provided as supporting information (Data S1 and S2).

### Ethics statement

The NC Division of Marine Fisheries gave permission to collect all *O. arbuscula* colonies used in this experiment (permit #706481).

## Results

### Effect of feeding on dry weight

**Figure 2:**
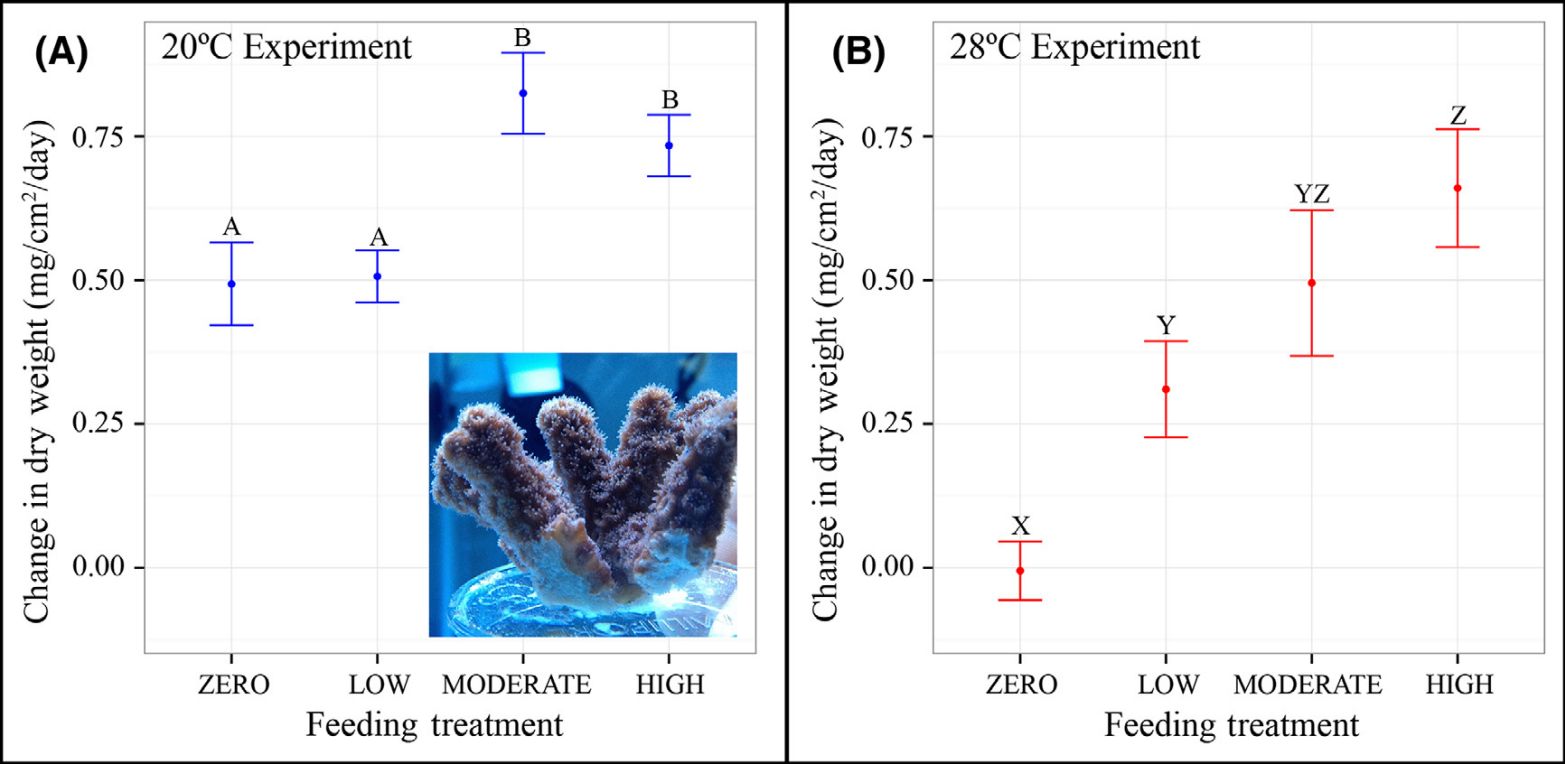
Effect of heterotrophy on *Oculina arbuscula* growth. (A) Change in dry weight per day across four feeding treatments in the 20°C thermal experiment. Error bars represent standard error. Letters represent statistical differences (A, B) as tested with Tukey's HSD. Inset illustrates an *Oculina arbuscula* fragment with polyps extended. (B) Change in dry weight per day across four feeding treatments in the 28°C thermal experiment. Error bars represent standard error. Letters represent statistical differences (X, Y, Z) as tested with Tukey's HSD.

Results revealed that feeding (*P* < 0.0001) significantly affected *O. arbuscula* calcification rates (expressed as dry weight; Table 1, Fig. 2) in both the mild and moderate stress experiments. Additionally, there was a main effect of genotype on calcification of *O. arbuscula* at both 20°C (*P* < 0.0001) and 28°C (*P* < 0.0001; Table 1). *Oculina arbuscula* fragments in the zero feeding treatment exhibited approximately zero net calcification at 28°C (moderate stress) while fragments reared at 20°C (mild stress) maintained positive net calcification. Calcification rates of moderate stress fragments in the zero feeding treatment were 0.50 ± 0.07 mg·cm^−2^·day^−1^ (±SE) less than mild stress specimens in zero feeding treatment (Fig. 2).

**Table 1:** Effect of feeding and genotype on dry weight and symbiont density

Variable	Factor	df	ss	*F*	*P*
**A. Mild stress experiment (20°C)**					
Dry weight	Feeding	3	1.4845	12.903	<0.0001
	Genotype	5	1.7445	9.098	<0.0001
	Feeding:Genotype	15	0.3691	0.642	0.8
log(symbiont density)	Feeding	3	1.849	2.302	0.09
	Genotype	5	3.647	2.725	0.03
	Feeding:Genotype	15	5.561	1.385	0.2
**B. Moderate stress experiment (28°C)**					
Dry weight	Feeding	3	4.396	20.615	<0.0001
	Genotype	5	6.082	17.113	<0.0001
	Feeding:Genotype	15	1.756	1.647	0.1
log(symbiont density)	Feeding	3	11.102	11.431	<0.0001
	Genotype	5	10.524	6.502	<0.001
	Feeding:Genotype	15	8.517	1.754	0.08

Results of a two‐factor ANOVA testing the effects of four feeding treatments and genotype on dry weight and symbiont density of *Oculina arbuscula* fragments. df, degrees of freedom; ss, sum of squares; *F* , *F*‐value; *P*,* P*‐value.

At 28°C, calcification of *O. arbuscula* fragments reared in the zero feeding treatment was significantly less than that of fragments reared in the low, moderate, and high feeding treatments (*P* = 0.005, *P* < 0.001, *P* < 0.001, respectively; Table 2B, Fig. 2B). Coral fragments reared in the low feeding treatment at 28°C also exhibited significantly lower calcification rates than fragments reared at high *Artemia* sp. nauplii concentrations (*P* = 0.002; Table 2B, Fig. 2B). Calcification rates of *O. arbuscula* fragments reared in the zero and low feeding treatments in the 20°C thermal experiment were significantly lower than fragments reared in the moderate and high feeding treatments at the same temperature (Table 2A, Fig. 2A).

**Table 2:** Pairwise comparisons of how feeding affected dry weight or symbiont density

Variable	Factor	Comparison	*P*
**A. Mild stress experiment (20°C)**			
Dry weight	Feeding	Zero‐low	0.997
		Zero‐moderate	<0.0001
		Zero‐high	0.004
		Low‐moderate	<0.0001
		Low‐high	0.006
		Moderate‐high	0.5
Symbiont density	Feeding	Zero‐low	0.2
		Zero‐moderate	1.0
		Zero‐high	1.0
		Low‐moderate	0.3
		Low‐high	0.09
		Moderate‐high	0.9
**B. Moderate stress experiment (28°C)**			
Dry Weight	Feeding	Zero‐low	0.005
		Zero‐moderate	<0.0001
		Zero‐high	0
		Low‐moderate	0.2
		Low‐high	0.002
		Moderate‐high	0.3
Symbiont density	Feeding	Zero‐low	0.04
		Zero‐moderate	0.0001
		Zero‐high	<0.0001
		Low‐moderate	0.2
		Low‐high	0.1
		Moderate‐high	1.0

Results of the Tukey's HSD tests evaluating the significance of the pairwise comparisons of factors that had a significant effect on dry weight or symbiont density of *Oculina arbuscula* fragments.

### Effect of feeding on symbiont density

**Figure 3:**
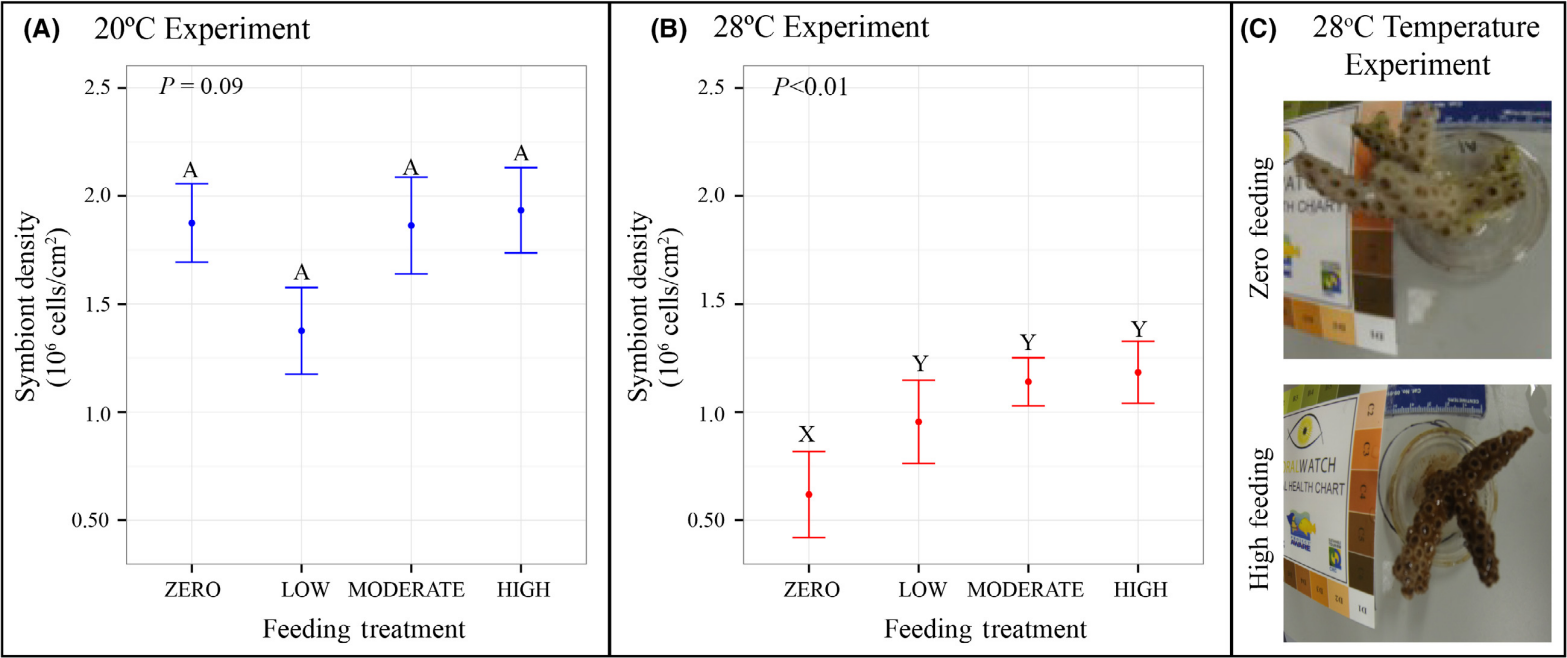
Effect of heterotrophy on *Oculina arbuscula* symbiont density. (A) Change in symbiont density per unit area (10^6^ cells·cm^−2^) across four feeding treatments in the 20°C thermal experiment. Error bars represent standard error. Letters represent statistical differences (A, B) as tested with Tukey's HSD. (B) Change in symbiont density per unit area (10^6^ cells·cm^−2^) across four feeding treatments in the 28°C thermal experiment. Error bars represent standard error. Letters represent statistical differences (X, Y) as tested with Tukey's HSD. (C) Images visualizing the effect of heterotrophy on *O. arbuscula* symbiont density. Bleaching was observed for this fragment that received no heterotrophic opportunity as compared to the same genotype that received high feeding in the thermal stress experiment.

In the moderate stress experiment, *O. arbuscula* fragments reared in the zero feeding treatment had significantly lower symbiont densities than fragments reared in the low, moderate, and high feeding treatments (*P* = 0.04, *P* = 0.001, *P* < 0.001, respectively; Table 2B, Fig. 3B). Feeding had no effect on symbiont density for *O. arbuscula* fragments reared in the mild thermal stress experiment (Table 1A, Fig. 3A). A main effect of genotype on symbiont density was also observed at 20°C (*P* = 0.03) and 28°C (*P* < 0.001; Table 1).

## Discussion

Our experiments reveal that the temperate coral *O. arbuscula* can utilize heterotrophic carbon to minimize growth reductions and loss of symbionts associated with exposure to moderate thermal stress. Because this species actively feeds, *O. arbuscula* may have an ecological advantage during thermal stress events that are predicted to occur with increasing frequency in future warmer oceans (Rhein et al. 2013). The facultative symbiosis of *O. arbuscula* is another potential mechanism that could allow the organism to deal with future climate change, as facultative symbioses are generally considered to offer flexibility to an organism in dealing with periods of rapid change (Kiers et al. 2010). This finding builds on previous studies that have demonstrated how heterotrophy can alleviate the negative effects of climate change, including rising temperature, OA, and the interactions of these two global scale stressors, for example (Grottoli et al. 2006; Borell et al. 2008; Towle et al. 2015).

### Heterotrophy mitigates loss of net calcification under temperature stress

Our study demonstrates that, when fed, *O. arbuscula* exhibited significantly greater net calcification under moderate thermal stress than when unfed (Fig. 2), suggesting that the negative effects of thermal stress can be mediated through heterotrophy. Unfed *O. arbuscula* fragments under moderate thermal stress were not able to maintain growth and exhibited approximately zero net calcification, an effect that was alleviated once corals were provided the opportunity for heterotrophy.

A recent study found that C acquired via heterotrophy was used for tissue building and skeletal growth in healthy, but not in bleached, colonies of the tropical corals *M. capitata* and *Porites compressa* (Hughes et al. 2010). In the temperate coral *O. arbuscula*, Leal et al. (2014) found that symbiotic colonies of this species obtained nutrition primarily from their endosymbionts, while aposymbiotic colonies depended primarily on heterotrophy of sediment organic matter as well as pico‐ and nanoplankton (<10 μm). This suggests that aposymbiotic colonies of *O. arbuscula* in the field likely depend almost entirely on heterotrophy, specifically on plankton <10 μm, for maintaining growth. Here, we studied symbiotic colonies of *O. arbuscula,* and results show that skeletal growth was maintained both with and without heterotrophic C under mild thermal stress. Notably, *O. arbuscula* under mild thermal stress grew significantly more when provided either moderate or high concentrations of *Artemia* sp. nauplii as compared to colonies reared with zero and low concentrations of food (Table 2A, Fig. 2A). Therefore, it is possible that *O. arbuscula* used symbiont‐derived C to maintain skeletal growth in the zero and low feeding treatments over the experimental interval at 20°C (Fig. 2). This agrees with Leal et al. (2014), who found that symbiotic colonies of *O. arbuscula* relied primarily on their endosymbionts for nutrition, regardless of the season.

### Temperature‐induced bleaching is mitigated by heterotrophy

Symbiont density was measured to determine the bleaching status of each *O. arbuscula* fragment as the symbionts of temperate (and tropical) corals provide photosynthetic carbon to the host, and the loss of those symbionts indicates holobiont stress and results in the loss of nutrients to the host (Miller 1995; Hoegh‐Guldberg 1999). While we cannot directly compare across thermal experiments, our data suggest that *O. arbuscula* had lower symbiont densities under moderate thermal stress when compared with corals reared under mild stress conditions (Fig. 3). Because *O. arbuscula* is facultatively symbiotic, it differs from most tropical corals in that prolonged bleaching does not necessarily indicate chronic stress or imminent death (Miller 1995); however, the loss of symbiont for this species could be indicative of stress and any loss of organic C input could have effects on holobiont fitness.

The results observed here contrast the findings of Rodolfo‐Metalpa et al. (2006), who found that symbionts of temperate symbiotic Mediterranean corals (*Cladocora caespitosa* and *O. patagonica*) were temperature tolerant, exhibiting no change in symbiont density or maximum quantum yield (*F*_v_ */F*_m_) under thermal stress up to 29°C. Other work has shown that algal symbiosis in another facultatively symbiotic coral, *Astrangia poculata*, is seasonally variable, with predicted chlorophyll density peaking in the late summer to early autumn and then decreasing to a relatively stable value throughout the winter (Dimond and Carrington 2007). Our findings suggest that thermal stress resulted in symbiont loss in the facultatively symbiotic temperate coral *O. arbuscula*. However, it is important to note that the observed decreases in symbiont density in the zero feeding treatment as compared to the other feeding treatments at 28°C could have resulted from the combination of temperature stress and a decline in food availability. The response we observed here*,* taken in consideration with previous studies on temperate corals, highlights that the responses of temperate corals to thermal stress are variable and possibly species specific.

Feeding did not affect symbiont density at 20°C; however, we observed that under moderate temperature stress, symbiont densities were greater in fed than unfed corals and as long as the fragments had some heterotrophic opportunity, symbiont density did not change significantly. These results highlight that heterotrophic feeding (at all concentrations) enabled *O. arbuscula* to maintain both significantly greater growth rates and symbiont densities as compared to corals in the zero feeding treatment under moderate thermal stress.

Our results confirm previous findings (Borell and Bischof 2008), which demonstrate heterotrophic food sources can reduce photophysiological damage in other coral species experiencing a thermal stress event. These authors proposed that in the absence of external food sources, temperature tolerance of *Stylophora pistillata* decreased because additional stressors (i.e., increased temperature) incur metabolic costs that limit physiological processes of the coral (Borell and Bischof 2008). Ferrier‐Pages et al. (2010) observed a similar result in three scleractinian coral species (*S. pistillata*,* Turbinaria reniformis* and *Galaxea fascicularis*): heterotrophic feeding reduced damage to the photosynthetic apparatus of symbionts and no bleaching was observed in fed, temperature‐stressed corals. Starved corals under temperature stress specifically demonstrated decreased electron transport and photosynthetic rates due to bleaching and resulting photoinhibition of photosystem II (Ferrier‐Pages et al. 2010). Therefore, it is possible that in our experiments, heterotrophic inputs prevented damage to *O. arbuscula* symbionts, possibly by bringing nitrogen and other essential nutrients to the coral holobiont, or by reducing the dependence of the coral host on the symbiont, thereby preventing bleaching.

### Implications for future climate change and management

Climate change, along with increases in human pressure on coastal ecosystems including population increases, industrialization, and agribusiness, will likely result in future increases of eutrophication in estuarine and coastal waters around the globe (Rabalais et al. 2009). Previous research and models suggest that eutrophication and climate change could increase primary production and phytoplankton standing stocks (Rabalais et al. 2009) and create a shift toward plankton communities of a smaller size class (Daufresne et al. 2009; Moran et al. 2010). For example, between 1979 and 2011, the northern Baltic Sea experienced a general increase in total phytoplankton biomass along with a decrease in total zooplankton abundance, leading researchers to conclude that the plankton community in this region shifted to favor plankton of smaller size classes as a result of warming and eutrophication (Suikkanen et al. 2013). These results have relevant implications for the NC coast and heterotrophic opportunity of *O. arbuscula*. Nutrient concentrations have increased in coastal NC waters since the end of World War II as a result of human activity (Paerl et al. 2006), and a recent study demonstrated preferential feeding of the coral on small‐size classes of phytoplankton (Leal et al. 2014). Collectively, these studies suggest that future climate change, which will likely result in thermal stress for *O. arbuscula* on coastal NC hard‐bottom habitats, could also provide an additional food source (i.e., increase in smaller plankton communities) to allow the coral to cope with that stress. These changes could particularly affect aposymbiotic colonies of *O. arbuscula,* which depend almost entirely on heterotrophy for inputs of organic carbon (Leal et al. 2014). It should be noted that even if the size class of plankton decreases in the future and if *O. arbuscula* is able to consume this readily available food source (Leal et al. 2014), this does not automatically indicate that the corals will be able to utilize this heterotrophic opportunity to mitigate the negative effects of climate change. In fact, our results suggest that even a doubling of available food source (represented by our high feeding treatment) would not result in a significant increase in *O. arbuscula* growth or symbiont density (Figs. 2, 3).

Conversely, several studies have demonstrated that climate change could result in future decreases in plankton concentrations (Roemmich and Mcgowan 1995; Richardson and Schoeman 2004). However, even if plankton concentrations decrease to half of their current values (represented by the low feeding treatment), our results indicate that *O. arbuscula* would still be able to utilize this heterotrophic opportunity to maintain growth and symbiont density at levels statistically equivalent to current‐day conditions (represented by the moderate feeding treatment) when exposed to thermal stress (Figs. 2, 3). Instead, only complete deprivation of *O. arbuscula* to heterotrophic opportunity under thermal stress is likely to result in significant decreases in fitness. Thus, under extreme circumstances in which zero plankton is available, these corals would essentially starve, making it difficult for *O. arbuscula* to maintain both growth rates and symbiont density under thermal stress. Our conclusion is supported by the significant decreases in both growth rates and symbiont density observed in the zero feeding treatment as compared to all other levels of heterotrophic opportunity in the moderate stress experiment (Figs. 2, 3).

It should be noted that feeding was not directly measured in this study; therefore, the impact of feeding is inferred based on the four concentrations of *Artemia* sp. nauplii provided in the experiment. It is possible that corals exposed to the different concentrations of *Artemia* sp. nauplii were not actually consuming significantly different amounts of food. This could potentially explain why there were no significant differences in coral growth or symbiont density observed between the moderate and high or moderate and low feeding treatments at 28°C (Figs. 2, 3). Additionally, the high feeding treatment in the 28°C experiment was 0.6°C warmer on average than the moderate feeding treatment at the same temperature (Table S1). This additional temperature stress could have prevented fragments in the high feeding treatment from exhibiting significantly greater growth rates and symbiont densities than fragments in the moderate feeding treatment at 28°C.

Our findings also indicate that *O. arbuscula* colonies varied in the degree to which heterotrophy will affect their response to future temperature stress. *Oculina arbuscula* genotype had a significant effect on growth and symbiont density in both thermal experiments (20 and 28°C; Table 1), which demonstrates within‐population variation in response to heterotrophy and temperature. This study investigated the responses of 12 *O. arbuscula* colonies from a single population; however, if these results are found to be consistent across populations and species, this suggests that significant genetic variation exists with respect to the influence of heterotrophy and temperature, perhaps providing fuel for natural selection.

Although *O. arbuscula* hard‐bottom ecosystems are less extensive in NC – the northern end of their range – their structural complexity provides arguably one of the most critical habitats in the region for an array of smaller organisms, which in turn supports a large diversity of recreationally and commercially relevant fish species (Deaton et al. 2010). Specifically, the snapper–grouper fisheries species that depend on hard‐bottom habitat (formed primarily by *O. arbuscula*) during migration produced a $3.6 million annual market between 1992 and 2001 (Deaton et al. 2010). However, these hard‐bottom ecosystems face threats from physical habitat loss/degradation (i.e., dredging and bottom‐disturbing fishing gear) and water quality degradation (i.e., nutrient enrichment and toxic chemical contamination), which will only increase as NC human populations grow (Deaton et al. 2010). Successful management of these habitats will aid in the maintenance of healthy fishery stocks for commercial and recreational use and conservation of the overall health of not only the NC coast, but across *O. arbuscula* habitats of the entire southeast US. A better understanding of how heterotrophy and SST warming interact to affect temperate corals, an important member of hard‐bottom ecosystems, will help to inform management decisions as SSTs will continue to increase throughout the century (Rhein et al. 2013).

## Conclusions

Although many studies have been conducted on the effects of climate change on coral reefs, few consider how heterotrophy interacts with a coral's response to climatic stressors, and even fewer consider this interaction in temperate species. This study aimed to understand the potentially beneficial impacts of coral heterotrophy in the context of ocean warming in a temperate scleractinian coral species. Results showed that heterotrophy provided thermally stressed corals with the necessary metabolic requirements to maintain growth and symbiont health/density. While it remains to be seen whether this response is conserved in other temperate and/or facultatively symbiotic coral species, the response observed here raises the question of how potential changes in nutrients and therefore planktonic communities could mitigate the negative effects of climate change on temperate reef ecosystems.

## Conflict of Interest

None declared.

## Supporting information


**Figure S1.** Buoyant weight‐dry weight correlation.

**Figure S2.** Data normality plots.

**Table S1**. Aquaria water quality.

**Data S1.** R script for data analysis.

**Data S2.** Growth and symbiont density data.
