# Supplementary figures and images for: Heterotrophy mitigates the response of the temperate coral Oculina arbuscula to temperature stress
Source: Ecol Evol. 2016 Aug 31;6(18):6758–69. doi: 10.1002/ece3.2399 (PMC5058543; doi:10.1002/ece3.2399)

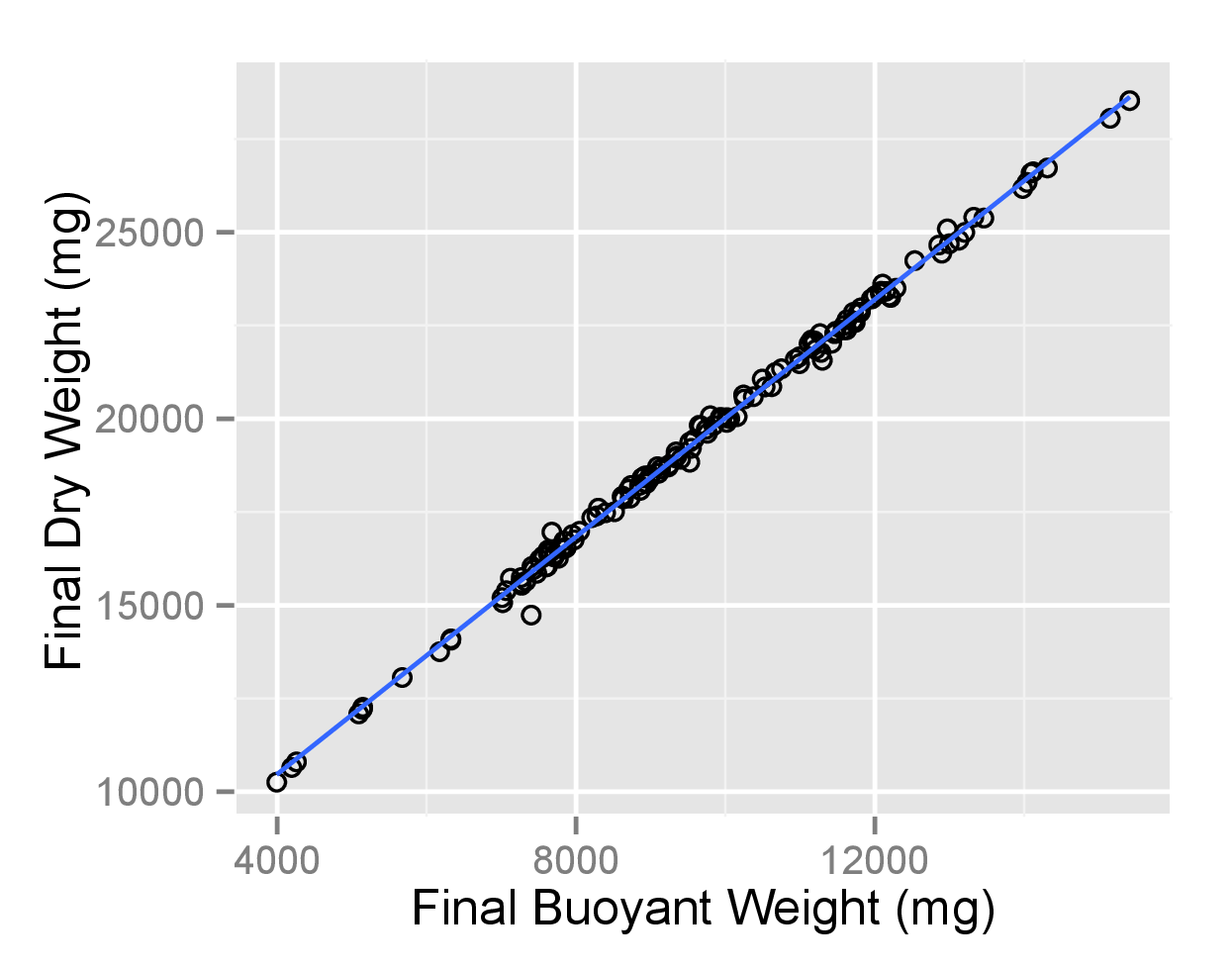

Supplement: Supplementary file 1 — Figure S1. Buoyant weight‐dry weight correlation. [file ECE3-6-6758-s001.tiff]

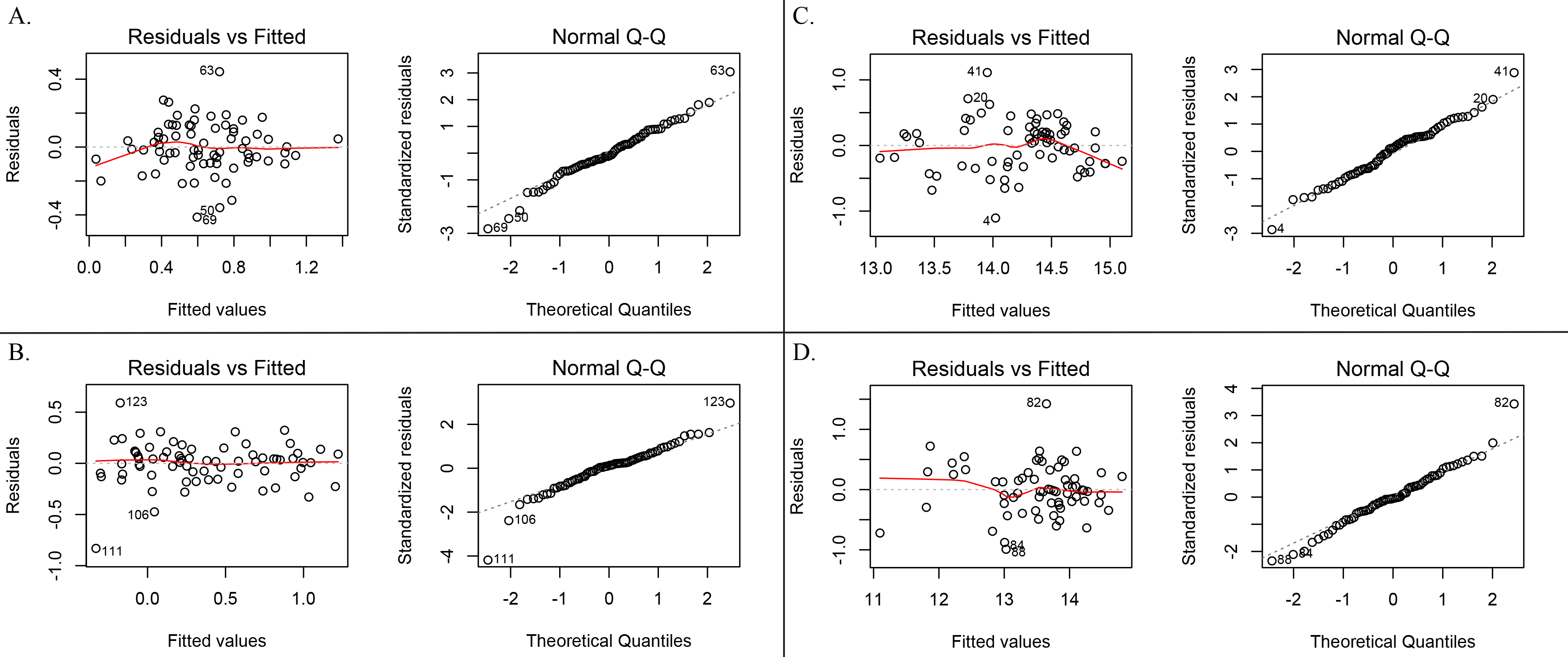

Supplement: Supplementary file 2 — Figure S2. Data normality plots. [file ECE3-6-6758-s002.tiff]
