# Supplementary material for: Heterotrophy mitigates the response of the temperate coral Oculina arbuscula to temperature stress
Source: Ecol Evol. 2016 Aug 31;6(18):6758–69. doi: 10.1002/ece3.2399 (PMC5058543; doi:10.1002/ece3.2399)
Supplement: Supplementary file 3 — Table S1. Aquaria water quality. [file ECE3-6-6758-s003.docx]

**S1 Table.** **Aquaria Water Quality.** Average measured water quality parameters for each experimental aquaria (n=12) during both temperature treatments. ‘SE’ is standard error, *n* is sample size.

| Control Temperature (20°C) | | | | | |
| --- | --- | --- | --- | --- | --- |
| Feeding |  | Zero | Low | Moderate | High |
| Temperature | °C | 20.1 | 20.3 | 20.1 | 20.4 |
|  | SE | 0.1 | 0.1 | 0.1 | 0.1 |
|  | Range | 19.9-20.9 | 20-21 | 19.9-20.7 | 20.1-21.2 |
|  | *n* | 48 | 48 | 48 | 48 |
| Salinity |  | 35.1 | 35.3 | 35.2 | 35.1 |
|  | SE | 0.1 | 0.1 | 0.1 | 0.1 |
|  | Range | 33.8-35.9 | 34.1-36.5 | 34.4-36 | 34.3-36 |
|  | *n* | 48 | 48 | 48 | 48 |
| *pH |  | 8.1 | 8.1 | 8.1 | 8.1 |
|  | SE | 0.1 | 0.1 | 0.1 | 0.1 |
|  | Range | 8.01-8.19 | 8.02-8.21 | 8.04-8.25 | 8.06-8.23 |
|  | *n* | 48 | 48 | 48 | 48 |
| Temperature Stress (28°C) | | | | | |
| Temperature | °C | 27.8 | 27.9 | 27.7 | 28.3 |
|  | SE | 0.1 | 0.1 | 0.1 | 0.1 |
|  | Range | 27-28.6 | 27-28.4 | 26.8-28.6 | 27.8-28.8 |
|  | *n* | 42 | 42 | 42 | 42 |
| Salinity |  | 36.2 | 36.9 | 38.0 | 37.7 |
|  | SE | 0.1 | 0.1 | 0.1 | 0.2 |
|  | Range | 34.3-39.1 | 35.6-39.3 | 36.7-39.5 | 35.6-39.5 |
|  | *n* | 42 | 42 | 42 | 42 |
| *pH |  | 8.2 | 8.2 | 8.2 | 8.2 |
|  | SE | 0.1 | 0.1 | 0.1 | 0.1 |
|  | Range | 8.02-8.32 | 8.08-8.35 | 8.01-8.32 | 8.07-8.31 |
|  | *n* | 42 | 42 | 42 | 42 |

*pH was measured using an Orion Star A211 pH meter with a ROSS Sure-Flow Combination pH probe calibrated with certified NBS pH buffers of 4.01, 7.00 and 10.01.
